# Forensic classification of gunpowder and fireworks powders by ATR-FT-IR spectroscopy and chemometric modelling

**DOI:** 10.3389/fchem.2026.1739998

**Published:** 2026-03-11

**Authors:** Abdulrahman Aljanaahi, Abdulla Aljanaahi, Noora Abdulkarim Ahli, Roudha Alblooshi, Abdulla Yasin, Iltaf Shah

**Affiliations:** 1 Dubai Police General Headquarters, Dubai, United Arab Emirates; 2 Department of Chemistry, College of Science, UAE University, Al Ain, United Arab Emirates

**Keywords:** energetic materials, explosives, machine learning, multivariate data analysis, preprocessing optimisation, spectral preprocessing

## Abstract

**Background:**

Rapid and reliable discrimination of ammunition propellants from consumer fireworks powders is critical in forensic explosives analysis but remains challenging due to overlapping chemical signatures and variability in formulations.

**Methods:**

In this study, attenuated total reflectance Fourier transform infrared (ATR-FT-IR) spectroscopy was combined with multivariate chemometric models to classify sixty-nine real-world gunpowder samples, including forty-three ammunition propellants and twenty-six fireworks powders. Several spectral preprocessing strategies, baseline correction, normalization, standard normal variate (SNV), and multiplicative scatter correction (MSC), were systematically evaluated to determine their effects on spectral variance and classification performance.

**Results:**

Principal component analysis (PCA) revealed that the main discriminant spectral regions correspond to nitrocellulose and nitroglycerin bands characteristic of propellants, and nitrate-perchlorate features typical of fireworks powders, confirming that the observed separation reflects genuine chemical differences. Linear discriminant analysis (LDA) achieved a classification accuracy of 97.1%, while support vector machine (SVM) models captured additional non-linear variance in the dataset. Regression-based approaches, including principal component regression (PCR), partial least squares regression (PLS-R), and support vector regression (SVR), indicated that apparent misclassifications were chemically plausible and largely attributable to compositional overlap rather than analytical error.

**Conclusions:**

The results demonstrate that both the selection and sequence of spectral preprocessing steps significantly influence model performance. The proposed ATR-FT-IR chemometric workflow provides a rapid, non-destructive, and interpretable screening approach for forensic laboratories and establishes a benchmark methodology for differentiating complex energetic materials.

## 1 Introduction

**FIGURE 1:**
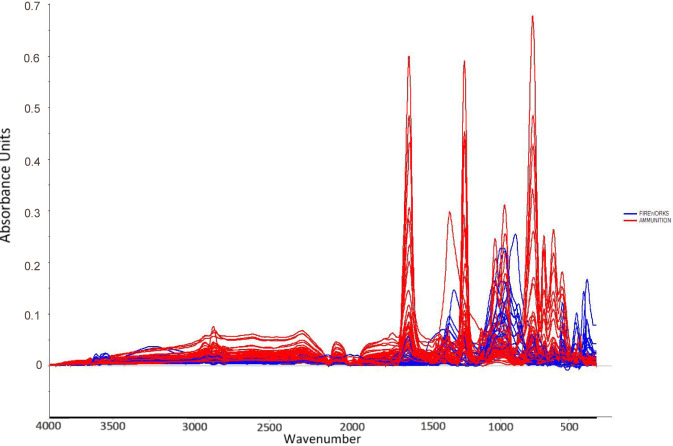
ATR-FT-IR spectra of the 69 gunpowder samples (43 ammunition propellants and 26 fireworks powders). Overlapping and distinct absorption features across the mid-infrared range (4000–400 cm^−1^) illustrate the spectral variability that motivates chemometric classification.

Explosive materials remain central to forensic investigations spanning criminal activity, terrorism, and accidental events, where rapid and reliable chemical identification underpins scene safety, attribution, and prosecution. In operational settings, investigators increasingly face chemically diverse formulations and post-blast residues that challenge conventional workflows, making fast, minimally destructive analytical tools a priority alongside confirmatory laboratory techniques (Forbes et al., 2020; Hutchinson et al., 2007; INTERPOL, 2019; Klapec et al., 2020; Sharma and Gadi, 2023; Zapata and García-Ruiz, 2021). Within this landscape, propellants (ammunition powders) and pyrotechnics (fireworks powders) are especially prevalent; yet their practical differentiation can be non-trivial because both are complex, multi-component mixtures whose signatures can overlap in routine analyses (López-López and García-Ruiz, 2014; Serol et al., 2023; Shrivastava et al., 2021; Zapata and García-Ruiz, 2021). As agencies and laboratories emphasize on-scene triage and robust laboratory confirmation, methods that couple rapid spectral screening with chemometric classification present a compelling path forward (Sleiman et al., 2016; van Damme et al., 2023; Zeman et al., 1993). The spectral variability motivating this approach is illustrated in Figure 1.

From a chemical standpoint, ammunition and fireworks powders are built on markedly different design principles. Modern smokeless propellants typically contain nitrocellulose (NC) as a polymeric energetic binder, often plasticized or co-formulated with nitroglycerin (NG) and stabilizers; their performance and long-term stability are sensitive to composition, aging, and storage (Trache and Tarchoun, 2019; Wang et al., 2025; Yao et al., 2022). By contrast, pyrotechnic formulations used in consumer fireworks generally rely on inorganic oxidizers (e.g., nitrates, perchlorates, sometimes sulfates) combined with fuels, binders, and metallic additives to achieve characteristic burn and visual effects (Banas et al., 2010; Ewing and Kazarian, 2017; Zapata and García-Ruiz, 2021). The heterogeneity intrinsic to these mixtures, particle size distributions, packing, additive packages, and potential environmental exposure, creates analytical challenges that manifest as spectral overlap, baseline variation, and between-batch variability (Arce-Rubí et al., 2020; Hu et al., 2021; López-López and García-Ruiz, 2014; Sun et al., 2022; Wolny et al., 2021; Zapata and García-Ruiz, 2021). These realities complicate univariate or library-matching approaches and argue for multivariate treatment to extract class-discriminating patterns reliably.

Vibrational spectroscopy, and Fourier transform infrared (FT-IR) spectroscopy in particular, has emerged as a mainstay in forensic analytics because it is rapid, minimally consumptive, and broadly accessible. Across explosives and residues, FT-IR and Raman have been used to query functional groups, binder signatures, and oxidizers, with multiple reviews consolidating their utility and limitations (Banas et al., 2020; López-López and García-Ruiz, 2014). Recent studies have further demonstrated the value of combining FT-IR with complementary techniques; for instance, infrared imaging has enhanced residue visualization on dark, patterned, and blood-contaminated substrates (Yüksel et al., 2019), while integrated chromophoric and instrumental approaches (SEM-EDS and FT-IR) have improved the objectivity of shooting distance estimation through multivariate statistical validation (Yuksel et al., 2025). Seminal FT-IR work demonstrated that multivariate analysis can separate post-blast residues and related materials more effectively than qualitative spectral inspection alone, especially when bands from nitrocellulose/nitroglycerin (NO_2_ stretches/bends) and inorganic oxidizers (nitrate ν_3_, perchlorate ν_3_/ν_4_) overlap or vary in intensity (Ewing and Kazarian, 2017; López-López and García-Ruiz, 2014). Moreover, advances in portable spectroscopy (notably near-infrared (NIR)) combined with multivariate models have enabled on-scene screening of intact explosives and residues, reducing turnaround time and informing subsequent sampling strategies (Sleiman et al., 2016; van Damme et al., 2023; Zeman et al., 1993). Nonetheless, fieldable systems face their own constraints (signal-to-noise, matrix variability, environmental interferences), further strengthening the case for robust preprocessing and chemometric modeling when the goal is reliable class-level discrimination rather than simple detection.

Chemometrics and machine learning provide the statistical backbone needed to transform rich, high-dimensional spectra into interpretable, decision-quality outputs. Authoritative reviews have charted their rise across forensic science, spanning Principal Component Analysis (PCA), Linear Discriminant Analysis (LDA), Partial Least Squares (PLS), Support Vector Machine (SVM), Support Vector Regression (SVR), and related algorithms, highlighting gains in classification accuracy, resilience to noise, and transparent feature attribution when models are well-calibrated and validated (Silva et al., 2019; Sauzier et al., 2020; dos Santos et al., 2010). Notably, the field has progressed from purely exploratory approaches such as PCA toward supervised classifiers (LDA, SVM) and regression-based strategies (PLS, SVR) that offer quantitative decision boundaries, with recent work increasingly adopting kernel-based and ensemble methods to handle the non-linear spectral variability inherent in complex forensic matrices. In the explosives domain specifically, multivariate approaches have improved the classification of residues, discrimination among formulations, and the integration of signals from modalities as diverse as Laser-induced breakdown spectroscopy, electrochemistry, and vibrational spectroscopy (Banas et al., 2010; Baumgarten et al., 2023; Castro-Suarez et al., 2017; Cetó et al., 2013; Chauhan et al., 2020; Zapata and García-Ruiz, 2021). Multi-instrumental strategies have also proven essential for characterizing modern ammunition components; elemental profiling of lead-free primers by inductively coupled plasma mass spectrometry (ICP-MS), scanning electron microscopy with energy-dispersive X-ray spectroscopy (SEM-EDS), and X-ray photoelectron spectroscopy (XPS) has revealed compositional variability that conventional criteria alone cannot resolve (Yüksel et al., 2023). Parallel successes across other forensic matrices, fibers (Alblooshi et al., 2024; Jalalvand, 2021), cosmetics (de Oliveira Neves et al., 2012), pharmaceuticals (Zhou et al., 2019), agro-food and near-infrared classification problems (Aljannahi et al., 2022; Rismiwandira et al., 2021), underscore the transferability of chemometric pipelines to complex mixtures encountered in casework. Recent domain-focused syntheses, including a comprehensive review specifically on analytical and chemometric strategies for explosives, further consolidate best practices and identify where systematic benchmarking is still needed (Aljanaahi et al., 2025).

Despite these advances, a critical gap remains: there are few systematic, head-to-head studies that (i) use attenuated total reflectance (ATR) FT-IR spectra from real-world ammunition and consumer fireworks powders, (ii) rigorously benchmark preprocessing pipelines, such as Standard Normal Variate (SNV), Multiplicative Scatter Correction (MSC), baseline correction, and normalization, and (iii) compare both linear and nonlinear algorithms under consistent validation schemes for explicit forensic discrimination. Previous works have examined post-blast residues or specific formulations (e.g., ammonium nitrate/fuel oil (ANFO), sparklers, nitrate-perchlorate systems) (Banas et al., 2010; Ewing and Kazarian, 2017; Schachel et al., 2020; D’Uva et al., 2020), yet a comprehensive evaluation of ATR-FT-IR combined with multi-pipeline machine learning for differentiating intact propellants and fireworks remains largely unexplored.

Accordingly, this study aims to fill this gap by constructing a comparative chemometric framework that systematically evaluates preprocessing order and model type across 69 real-world samples (43 ammunition propellants and 26 fireworks powders). The research advances the field by linking spectral-chemical variance to model behavior, providing an interpretable, deployable workflow for forensic explosives laboratories.

## 2 Materials and methods

### 2.1 Samples and safety

Sixty-nine gunpowder samples were examined: forty-three smokeless propellant powders recovered from ammunition (handgun and rifle cartridges, shotgun shells, and selected ordnance charges) and twenty-six pyrotechnic powders collected from consumer fireworks such as aerial shells, firecrackers, fountains, and multi-effect devices. This distribution was selected to reflect the chemical heterogeneity typical of nitrocellulose-based propellants versus oxidizer-rich pyrotechnic formulations observed in recent forensic studies (Serol et al., 2023; Shrivastava et al., 2021).

Sampling and handling were conducted by explosives specialists of Dubai Police under institutional protocols aligned with international safety guidance for explosives work (INTERPOL, 2019; Klapec et al., 2020). All extractions used non-sparking tools on antistatic surfaces behind protective barriers. Powders were transferred into inert, airtight vials, sealed, labeled with unique identifiers, and logged for full chain-of-custody traceability. Samples were stored in climate-controlled magazines until analysis. Contamination control procedures, including nitrile gloves, disposable tools, and antistatic mats, were maintained throughout. Residual material and waste were neutralized and disposed of according to explosives-safety regulations (INTERPOL, 2019; Klapec et al., 2020).

### 2.2 FT-IR spectral acquisition

Spectra were collected using a Bruker FT-IR spectrometer equipped with a diamond attenuated-total-reflectance (ATR) accessory. For each specimen, the crystal contact pressure was kept constant, and the crystal was cleaned with solvent and lint-free tissue between measurements. Spectral windows were recorded from 4000 to 400 cm^−1^ at 4 cm^−1^ resolution with 100 co-added scans per spectrum. Background spectra were acquired under identical conditions immediately before each run. Raw data were saved in OPUS format and imported directly into Aspen Unscrambler® X for preprocessing and modeling.

The selection of ATR-FT-IR is consistent with established forensic applications for identifying energetic materials, including nitrocellulose- and nitrate-based propellants and post-blast residues (Banas et al., 2010; Ewing and Kazarian, 2017; López-López and García-Ruiz, 2014). These techniques have demonstrated excellent reproducibility, minimal sample preparation, and non-destructive performance in explosives analysis.

### 2.3 Spectral preprocessing

Spectral preprocessing was applied to mitigate baseline drift, particle-size scatter, and other matrix effects that can obscure diagnostic absorption bands. Operations included SNV scaling, MSC, baseline correction, vector normalization, and Savitzky-Golay smoothing. Various operation sequences were evaluated, including MSC alone, SNV alone, baseline followed by normalization, baseline followed by MSC, baseline followed by normalization followed by SNV, MSC followed by SNV, and baseline alone, to determine their influence on model performance.

Each operation targets a distinct source of spectral distortion. Baseline correction removes low-frequency drift caused by instrumental electronics or sample-surface coupling at the ATR crystal interface. Normalization scales spectra to a common intensity range, compensating for differences in applied pressure or powder-packing density. SNV centres and scales each spectrum individually, reducing multiplicative scatter introduced by particle-size variation and surface roughness. MSC achieves a similar scatter correction but does so relative to a reference (mean) spectrum, making it sensitive to the overall dataset structure. The order in which these operations are applied matters because each transformation alters the variance available to subsequent steps; for example, applying MSC before SNV may over-correct scatter if both target similar multiplicative effects, whereas baseline correction followed by normalization preserves the relative band intensities needed for class discrimination. By evaluating multiple sequences rather than a single fixed pipeline, this study explicitly tests whether preprocessing order introduces systematic bias into downstream classification and regression models.

Pipeline effects were first screened via PCA to assess cluster compactness and noise reduction, then propagated to supervised models. The use of these preprocessing approaches follows best-practice recommendations in forensic chemometrics, where variance stabilization and scatter correction are prerequisites for accurate classification (Silva et al., 2019; Sauzier et al., 2020; dos Santos et al., 2010).

### 2.4 Chemometric and machine learning models

Exploratory structure within the dataset was investigated using PCA to visualize variance patterns and detect potential outliers before supervised learning (Sauzier et al., 2020; Silva et al., 2019). For categorical discrimination between ammunition and fireworks, LDA and SVM classifiers were trained with linear, polynomial, Radial Basis Function (RBF), and sigmoid kernels. For regression-based profiling, PCR, PLS-R, and SVR were implemented.

Model hyperparameters, including the regularization constant (C) and kernel width (γ) for RBF kernels and polynomial degree for polynomial kernels, were optimized through inner k-fold cross-validation. These approaches are well-established in forensic spectroscopic applications (Banas et al., 2010; Castro-Suarez et al., 2017; Cetó et al., 2013; Jalalvand, 2021; Sauzier et al., 2020; Silva et al., 2019).

### 2.5 Validation strategy and performance metrics

Data were partitioned into stratified training (70%) and validation (30%) subsets, a ratio widely adopted in chemometric studies of small-to-moderate forensic datasets where it balances sufficient model training with a meaningful independent test (Sauzier et al., 2020; Silva et al., 2019). Given that the dataset comprises 69 samples, a larger test fraction would reduce the training pool below the minimum needed for stable cross-validated hyperparameter selection, while a smaller fraction would yield a validation set too small for reliable accuracy estimation. Within the training partition, repeated k-fold cross-validation was employed for model selection and hyperparameter tuning to prevent optimistic bias; final models were refit on the complete training set and evaluated once on the untouched validation subset. Classification models were assessed by overall accuracy and confusion matrices, with class-specific sensitivity and specificity. Regression models were evaluated by coefficient of determination (R^2^), root-mean-square error (RMSE), and calibration/validation slope and offset. R^2^ quantifies the proportion of variance in the response variable explained by the model and is defined as:

$$R^{2}=1-\left(\text{SS}\_\text{res }/\text{ SS}\_\text{tot}\right)=1-\left[\Sigma {\left(yᵢ-ŷᵢ\right)}^{2}\;/\;\Sigma {\left(yᵢ-ȳ\right)}^{2}\right]$$

where yᵢ is the observed value, ŷᵢ is the predicted value, and ȳ is the mean of observed values. RMSE measures the average magnitude of prediction error in the original units and is calculated as:

$$\text{RMSE}=\sqrt{\left[\left(1/n\right)\;\Sigma {\left(yᵢ-ŷᵢ\right)}^{2}\right]}$$

where n is the number of observations. Together, R^2^ and RMSE provide complementary assessments of model fit: R^2^ captures explained variance on a normalised scale, while RMSE reflects absolute prediction error and is sensitive to outliers.

Potential outliers identified by PCA score-distance or Hotelling’s T^2^ were examined for experimental causes, and sensitivity analyses were performed with and without these data points. The validation framework and statistical metrics follow chemometric recommendations for forensic vibrational-spectroscopy workflows (Sauzier et al., 2020; Silva et al., 2019).

**FIGURE 2:**
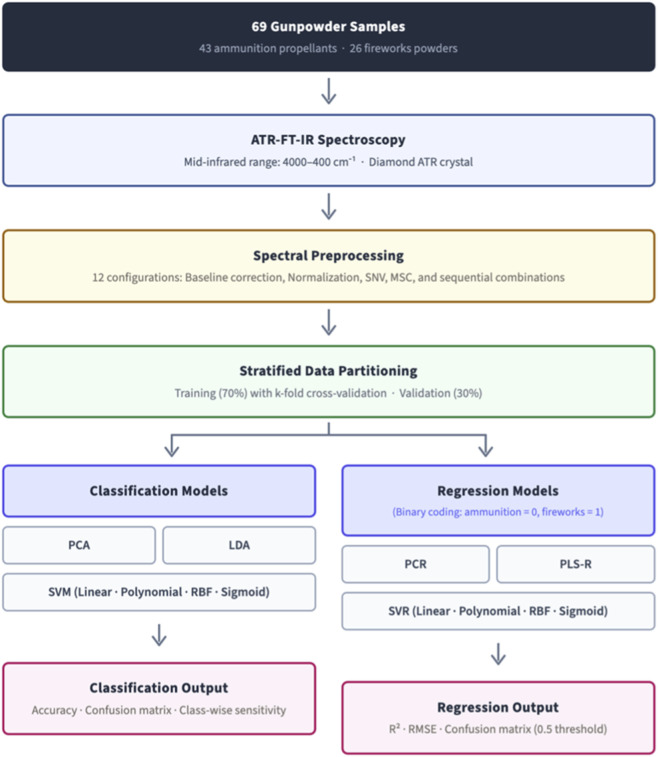
Schematic overview of the analytical workflow. Sixty-nine gunpowder samples (43 ammunition propellants, 26 fireworks powders) were analysed by ATR-FT-IR spectroscopy and subjected to 12 preprocessing configurations. Preprocessed spectra were partitioned into stratified training (70%) and validation (30%) subsets. Classification models (PCA, LDA, SVM) and regression models (PCR, PLS-R, SVR) were evaluated in parallel, with regression outputs converted to classification via a 0.5 decision threshold.

The complete analytical workflow is summarised in Figure 2. Ethics and safety statement: all experimental work involving energetic or pyrotechnic materials complied with institutional approvals and internationally recognized explosives-handling standards (INTERPOL, 2019; Klapec et al., 2020).

## 3 Results

### 3.1 Representative spectra and diagnostic band identification

**FIGURE 3:**
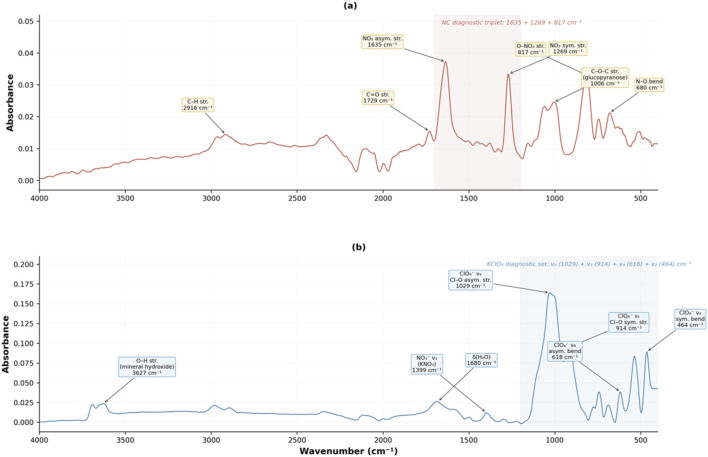
Representative ATR-FT-IR spectra of **(a)** Sample 9, an ammunition propellant (smokeless powder) showing the nitrocellulose diagnostic triplet (1635, 1269, and 817 cm^−1^) and additional organic functional group absorptions, and **(b)** Sample 48, a fireworks powder (pyrotechnic mixture) showing the potassium perchlorate diagnostic set (1029, 914, 618, and 464 cm^−1^), potassium nitrate (1399 cm^−1^), mineral hydroxide O-H stretch (3627 cm^−1^), and water bending mode (1680 cm^−1^).

To illustrate the spectral basis for class discrimination, Figure 3 presents representative ATR-FT-IR spectra for one ammunition propellant (Sample 9, smokeless powder) and one fireworks powder (Sample 48, pyrotechnic mixture). The ammunition spectrum is dominated by absorption features characteristic of nitrocellulose. The asymmetric and symmetric stretching vibrations of the nitro group (NO_2_) appear at 1635 cm^−1^ and 1269 cm^−1^, respectively, while the O-NO_2_ stretching vibration is observed at 817 cm^−1^; together, these three bands form a diagnostic triplet for nitrocellulose (Larsson, 2014; Nunes et al., 2020). Additional features include C-H stretching at 2916 cm^−1^, C=O stretching at 1729 cm^-1^ attributable to ester carbonyls from plasticizers or degradation products, C-O-C stretching from the glucopyranose ring of cellulose at 1006 cm^−1^ (Nunes et al., 2020), and N-O bending at 680 cm^−1^ (Huang et al., 2023).

The fireworks spectrum exhibits a markedly different profile, dominated by inorganic oxidizer signatures. The asymmetric stretching vibration of the nitrate ion (NO_3_^−^ ν_3_) from potassium nitrate (KNO_3_) appears at 1399 cm^−1^. A complete set of fundamental vibrational modes of the perchlorate ion (ClO_4_^−^) from potassium perchlorate (KClO_4_) is observed: the asymmetric stretch (ν_3_) at 1029 cm^−1^, symmetric stretch (ν_1_) at 914 cm^−1^, asymmetric bend (ν_4_) at 618 cm^−1^, and symmetric bend (ν_2_) at 464 cm^−1^ (Holló et al., 2019). An O-H stretching band at 3627 cm^−1^ indicates the presence of a mineral hydroxide, and a band at 1680 cm^−1^ corresponds to the bending mode of water (δ(H_2_O)), consistent with adsorbed moisture or hydrated mineral phases.

These contrasting spectral profiles confirm that the two powder classes are built on fundamentally different chemistries: organic nitro-ester functionalities in ammunition propellants versus inorganic nitrate and perchlorate salts in fireworks powders. This chemical distinction provides the physical basis for the chemometric separation explored in the following sections.

### 3.2 PCA

PCA was applied to examine the intrinsic variance among the 69 gunpowder spectra and to evaluate the influence of different spectral preprocessing strategies on sample clustering. Several preprocessing combinations were investigated, including MSC, SNV, normalization, and baseline correction applied in different sequences. These configurations comprised MSC alone, SNV alone, normalization followed by SNV, MSC followed by SNV, baseline correction followed by normalization and SNV, and baseline correction followed by MSC.

**FIGURE 4:**
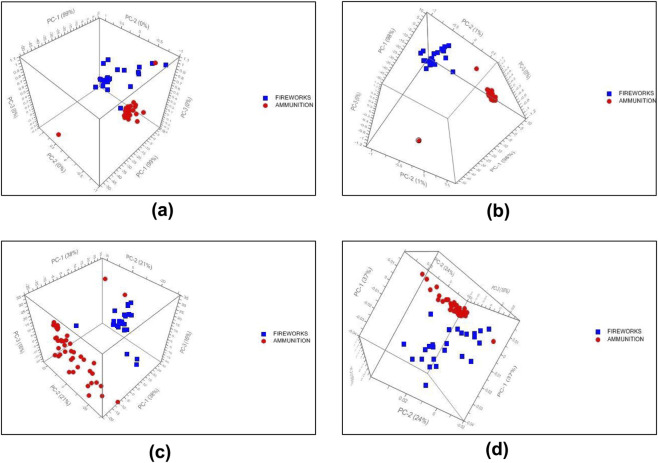
3D PCA score plots of 69 gunpowder samples (43 ammunition, red circles; 26 fireworks, blue squares), illustrating the effect of preprocessing on class clustering. Variance explained by each principal component is indicated on the axes. **(a)** MSC preprocessing (PC-1: 99%, PC-2: less than 1%, PC-3: less than 1%). **(b)** Baseline correction followed by MSC (PC-1: 98%, PC-2: 1%, PC-3: less than 1%), yielding the most distinct class separation. **(c)** MSC followed by SNV (PC-1: 38%, PC-2: 21%, PC-3: 16%). **(d)** Normalization (PC-1: 37%, PC-2: 24%, PC-3: 16%).

The clustering behavior varied noticeably among these preprocessing approaches, as illustrated by the variance explained by the first three principal components in each configuration (Figure 4). When MSC was applied as a single step (Figure 4a), PC-1 captured 99% of the total variance with negligible contributions from PC-2 and PC-3 (both less than 1%). Despite this high concentration of variance in a single component, the resulting score plot exhibited broad overlap between ammunition and fireworks samples, indicating that the dominant spectral variance under MSC correction alone reflects intensity-scaling differences rather than class-discriminating chemical features.

In contrast, the application of baseline correction followed by MSC (Figure 4b) produced the most distinct class separation, despite a similar variance distribution (PC-1: 98%, PC-2: 1%, PC-3: less than 1%). The prior removal of low-frequency baseline drift evidently allowed MSC to correct scatter effects without conflating them with chemically meaningful variation, enabling the first principal component to capture class-relevant variance. Ammunition samples formed a tight and homogeneous cluster, while fireworks samples were more widely distributed, reflecting their broader compositional variability in oxidizer and fuel content. One ammunition sample was projected away from the main ammunition cluster, suggesting possible compositional similarity with certain pyrotechnic formulations.

When MSC was followed by SNV (Figure 4c), the variance was distributed more broadly across components (PC-1: 38%, PC-2: 21%, PC-3: 16%, cumulative 75%), and both classes showed wider scatter with partial overlap. A similar pattern was observed under normalization alone (Figure 4d; PC-1: 37%, PC-2: 24%, PC-3: 16%, cumulative 77%), where ammunition and fireworks samples occupied distinct but proximate regions of the score space. The more even variance distribution in these configurations suggests that the combination of MSC and SNV, or normalization alone, redistributes spectral variance across multiple components rather than concentrating class-discriminating information in the first component.

**FIGURE 5:**
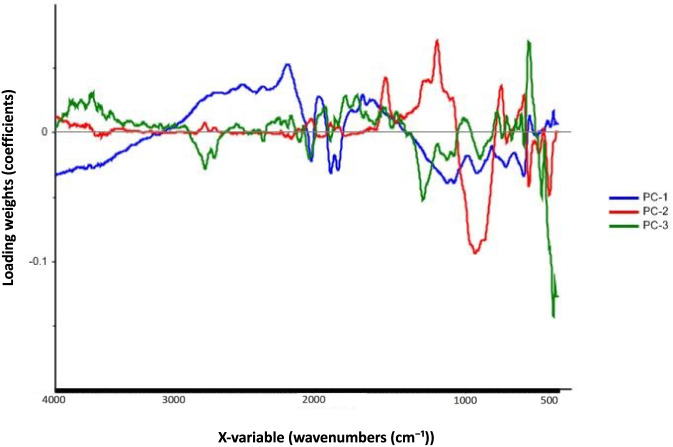
Loadings plot for the baseline correction followed by MSC preprocessed model, corresponding to the score plot in Figure 4B.

The corresponding loading vectors for the optimized baseline followed by MSC model (Figure 5) indicated that wavenumber regions near 2400 cm^−1^, 1500 cm^−1^, and 700 cm^−1^ contributed most strongly to the observed separation. These bands are characteristic of nitrate, nitrocellulose, and carbonate groups, respectively, which are key constituents in many propellant and pyrotechnic formulations. These loading patterns are consistent with the diagnostic bands identified in the representative spectra (Figure 3), where the nitrocellulose triplet (1635, 1269, 817 cm^−1^) and perchlorate/nitrate features (1029, 914, 618, 464, 1399 cm^−1^) were the primary distinguishing signatures. Similar discriminating features have been reported in previous FT-IR investigations of energetic materials, supporting the chemical validity of the observed variance (Banas et al., 2010; Ewing and Kazarian, 2017; López-López and García-Ruiz, 2014).

### 3.3 LDA

LDA was applied to classify the FT-IR spectra of gunpowder samples into their respective categories of ammunition and fireworks. The analysis aimed to evaluate how different preprocessing strategies influenced class separation and predictive accuracy. Twelve preprocessing configurations were tested, combining MSC, SNV, normalization, and baseline correction in different sequential orders.

LDA performance was highly dependent on the preprocessing method applied. Among all configurations, the highest classification accuracy of 97.1% was obtained using SNV alone, as well as using sequential pipelines of normalization followed by SNV and baseline correction followed by normalization and SNV. These techniques effectively standardized variance and minimized baseline shifts, thereby enhancing spectral clarity and inter-class separation. In contrast, the configuration in which SNV was followed by normalization produced the lowest accuracy (53.6%), demonstrating that an inappropriate order of preprocessing steps can amplify noise and reduce the discriminant power of the model. The retention of this low-performing configuration in the analysis is deliberate: it provides direct empirical evidence that preprocessing order, not merely preprocessing selection, is a critical variable in chemometric workflows, a finding with practical implications for forensic laboratories developing standard operating procedures.

**FIGURE 6:**
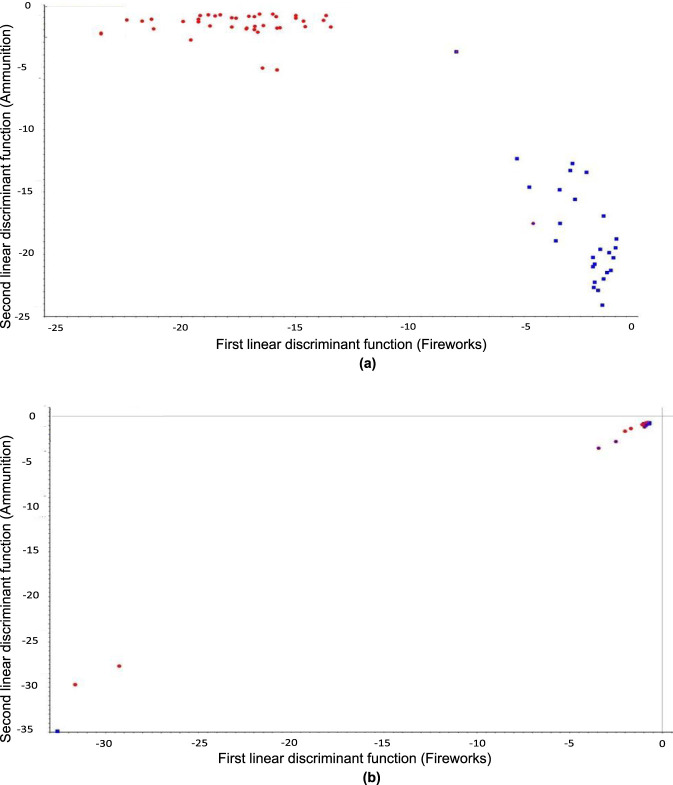
LDA classification of gunpowder samples (ammunition in red, fireworks in blue): **(a)** high-accuracy model after SNV preprocessing (97.1% accuracy); **(b)** low-accuracy model after SNV followed by normalization (53.6% accuracy), retained to demonstrate the critical effect of preprocessing order on classification performance.

The optimized model employing SNV preprocessing (Figure 6a) yielded compact and well-separated clusters for ammunition and fireworks, each represented by tight, internally consistent groupings. Most misclassifications were limited to one sample per category, primarily at the boundary between clusters. This high level of performance indicates that SNV preprocessing efficiently corrects for multiplicative scatter and intensity scaling, allowing the LDA algorithm to emphasize chemically meaningful variance. Conversely, the model constructed after applying SNV followed by normalization (Figure 6b) showed substantial overlap between the two classes, illustrating how preprocessing order directly affects discriminant quality and accuracy.

To provide a more detailed assessment of classification performance beyond overall accuracy, class-wise sensitivity was calculated for each preprocessing configuration (Table 1). Sensitivity quantifies the proportion of correctly classified samples within each class, revealing asymmetries that overall accuracy alone may mask. For the best-performing configurations (SNV alone, normalization followed by SNV, and baseline followed by normalization followed by SNV), ammunition sensitivity reached 97.7% (42 of 43 correctly classified) and fireworks sensitivity reached 96.2% (25 of 26 correctly classified). In contrast, the SNV followed by normalization configuration reduced fireworks sensitivity to 50.0% and ammunition sensitivity to 55.8%, confirming that both classes were severely affected by the inappropriate preprocessing order. Configurations such as MSC followed by baseline and baseline only achieved 100% ammunition sensitivity but substantially lower fireworks sensitivity (73.1%), indicating a class-dependent bias toward the majority class when preprocessing inadequately resolves inter-class spectral differences.

**TABLE 1:** LDA classification performance across preprocessing configurations, showing overall accuracy, misclassification counts, and class-wise sensitivity for both fireworks (n = 26) and ammunition (n = 43) samples.

Preprocessing technique	Accuracy (%)	Misclassified fireworks	Misclassified ammunition	Fireworks sensitivity (%)	Ammunition sensitivity (%)
No preprocessing	88.4	7	1	73.1	97.7
MSC only	95.65	1	2	96.2	95.3
SNV only	97.1	1	1	96.2	97.7
Normalization only	94.2	3	1	88.5	97.7
Normalization → SNV	97.1	1	1	96.2	97.7
SNV → normalization	53.62	13	19	**50.0**	**55.8**
MSC → SNV	95.65	1	2	96.2	95.3
MSC → baseline	89.86	7	0	73.1	100.0
Baseline → normalization	94.2	3	1	88.5	97.7
Baseline → normalization → SNV	97.1	1	1	96.2	97.7
Baseline → MSC	95.65	1	2	96.2	95.3
Baseline only	89.86	7	0	73.1	100.0

The dominance of the SNV-based configuration is consistent with previous FT-IR chemometric studies of energetic and polymeric materials, where standard normal variate preprocessing was shown to significantly improve class separability by removing path-length and scattering artifacts (Banas et al., 2010; Ewing and Kazarian, 2017; López-López and García-Ruiz, 2014). The high predictive stability across several linear preprocessing sequences also supports the robustness of LDA for forensic discrimination tasks, aligning with findings that optimized preprocessing pipelines can transform subtle spectral differences into reliable classification boundaries (Silva et al., 2019).

### 3.4 SVM

SVM algorithms were applied to the FT-IR dataset to further evaluate the capacity of nonlinear classifiers to discriminate between ammunition and fireworks powders. Four kernel functions, linear, polynomial, RBF, and sigmoid, were tested using spectra preprocessed with multiple configurations, including single-step and sequential combinations of MSC, SNV, normalization, and baseline correction.

The classification accuracy varied markedly depending on both the preprocessing strategy and the kernel employed. The RBF kernel, when applied to spectra preprocessed using baseline correction followed by normalization and SNV, achieved the best performance with a validation accuracy of 97.1%. Under the same preprocessing conditions, the sigmoid kernel also achieved comparable accuracy, whereas the polynomial kernel consistently underperformed across all configurations. The linear kernel demonstrated stable yet slightly lower accuracy, performing best with SNV-preprocessed spectra, where it yielded a perfect training accuracy of 100% and validation accuracy of 94.2%.

The influence of preprocessing was pronounced across all kernel types. Configurations incorporating SNV, either alone or as part of the baseline followed by normalization followed by SNV sequence, consistently enhanced class discrimination by reducing multiplicative scatter effects and improving baseline stability. In contrast, models built on unprocessed or poorly ordered sequences (such as SNV followed by normalization) displayed greater intra-class overlap and elevated misclassification rates, particularly among the fireworks samples. This variability reflects the inherent compositional heterogeneity of pyrotechnic mixtures and underscores the necessity of preprocessing optimization prior to SVM modeling.

The polynomial kernel exhibited two distinct failure modes under suboptimal preprocessing conditions (Table 2). In several configurations (MSC followed by baseline, normalization only, baseline only, and baseline followed by normalization), the polynomial kernel misclassified all 26 fireworks samples while correctly identifying all ammunition samples, yielding a validation accuracy of approximately 62%. This pattern indicates that the model defaulted to predicting the majority class (ammunition) for all inputs, effectively failing to learn the class boundary. Conversely, under MSC only and baseline followed by MSC preprocessing, the polynomial kernel misclassified 42 of 43 ammunition samples while correctly classifying all fireworks samples (validation accuracy approximately 62%). In this reverse failure mode, the model collapsed toward predicting the minority class. Both patterns reflect the polynomial kernel’s sensitivity to the high dimensionality and spectral variance structure of the dataset; when preprocessing does not adequately reduce scatter and baseline effects, the kernel function maps the data into a feature space where one class dominates, preventing meaningful discrimination. In contrast, the RBF kernel exhibited the strongest adaptability to nonlinearities, enabling improved generalization and lower misclassification across diverse preprocessing combinations. This finding is consistent with previous studies demonstrating that RBF kernels are well suited for forensic spectral classification tasks involving explosives and chemically diverse residues (Aljanaahi et al., 2025; Banas et al., 2010; Silva et al., 2019; Sleiman et al., 2016).

**TABLE 2:** SVM classification performance across kernel functions and preprocessing configurations, showing training and validation accuracies, misclassification counts, and class-wise sensitivity for fireworks (n = 26) and ammunition (n = 43) samples.

Preprocessing technique	Kernel	Training accuracy (%)	Validation accuracy (%)	Misclassified fireworks	Misclassified ammunition	Fireworks sensitivity (%)	Ammunition sensitivity (%)
No preprocessing	Sigmoid	81.16	92.8	13	0	50.0	100.0
MSC only	Sigmoid	95.65	88.44	1	2	96.2	95.3
MSC only	Linear	97.1	94.2	1	1	96.2	97.7
MSC only	RBF	97.1	92.8	1	1	96.2	97.7
MSC only	Polynomial	39.13	62.3	0	42	100.0	2.3
SNV only	Linear	100.0	94.2	0	0	100.0	100.0
SNV only	Sigmoid	97.1	97.1	1	1	96.2	97.7
SNV only	RBF	97.1	97.1	1	1	96.2	97.7
SNV only	Polynomial	97.1	97.1	1	1	96.2	97.7
MSC → SNV	Sigmoid	95.65	95.65	1	2	96.2	95.3
MSC → SNV	Linear	100.0	89.9	0	0	100.0	100.0
MSC → SNV	RBF	97.1	95.65	1	1	96.2	97.7
MSC → SNV	Polynomial	95.65	94.2	1	2	96.2	95.3
MSC → baseline	Polynomial	62.32	62.31	26	0	0.0	100.0
MSC → baseline	Linear	97.1	94.2	1	1	96.2	97.7
MSC → baseline	Sigmoid	62.32	63.77	26	0	0.0	100.0
MSC → baseline	RBF	95.65	94.2	2	1	92.3	97.7
Normalization only	Linear	97.1	97.1	1	1	96.2	97.7
Normalization only	Polynomial	62.32	62.31	26	0	0.0	100.0
Normalization only	RBF	97.1	95.65	1	1	96.2	97.7
Normalization only	Sigmoid	65.22	60.87	24	0	7.7	100.0
Normalization → SNV	Sigmoid	97.1	97.1	1	1	96.2	97.7
Normalization → SNV	Linear	100.0	94.2	0	0	100.0	100.0
Normalization → SNV	Polynomial	97.1	97.1	1	1	96.2	97.7
Normalization → SNV	RBF	97.1	97.1	1	1	96.2	97.7
Baseline only	Linear	97.1	92.75	1	1	96.2	97.7
Baseline only	Polynomial	62.32	62.31	26	0	0.0	100.0
Baseline only	RBF	81.16	91.3	12	1	53.8	97.7
Baseline only	Sigmoid	82.61	91.3	12	0	53.8	100.0
Baseline → normalization	Sigmoid	97.1	97.1	1	1	96.2	97.7
Baseline → normalization	Linear	97.1	97.1	1	1	96.2	97.7
Baseline → normalization	Polynomial	62.32	62.31	26	0	0.0	100.0
Baseline → normalization	RBF	98.55	95.65	0	1	100.0	97.7
Baseline → normalization → SNV	RBF	97.1	97.1	1	1	96.2	97.7
Baseline → normalization → SNV	Sigmoid	97.1	97.1	1	1	96.2	97.7
Baseline → normalization → SNV	Linear	100.0	94.2	0	0	100.0	100.0
Baseline → normalization → SNV	Polynomial	97.1	97.1	1	1	96.2	97.7
Baseline → MSC	Linear	97.1	94.2	1	1	96.2	97.7
Baseline → MSC	Polynomial	39.13	62.31	0	42	100.0	2.3
Baseline → MSC	RBF	97.1	92.75	1	1	96.2	97.7
Baseline → MSC	Sigmoid	95.65	88.41	1	2	96.2	95.3

Class-wise sensitivity analysis (Table 2) further illustrates the asymmetric impact of kernel-preprocessing combinations on each class. For the best-performing configurations (RBF and sigmoid kernels with SNV-based preprocessing), both ammunition sensitivity and fireworks sensitivity exceeded 96%, confirming balanced discrimination. However, the polynomial kernel failure modes described above produced extreme asymmetry: 0% sensitivity for the class being entirely misclassified, versus 100% for the other. These results reinforce that overall accuracy alone can mask severe class-specific deficiencies, and that sensitivity reporting for each class is essential when evaluating forensic classification models operating on imbalanced datasets.

Table 2 summarizes the detailed training and validation accuracies, along with misclassification counts and class-wise sensitivity, for each kernel-preprocessing combination. The results indicate that SNV-based preprocessing and RBF kernel modeling provide the most balanced performance, combining high predictive accuracy with minimal overfitting. Such outcomes align with established chemometric principles that emphasize the synergy between appropriate spectral correction and nonlinear classification in achieving robust discrimination of forensic materials (Banas et al., 2010; López-López and García-Ruiz, 2014; Silva et al., 2019).

### 3.5 PCR

PCR was applied to model the relationship between the FT-IR spectral data and sample classification, linking latent spectral variance to the categorical differentiation between ammunition and fireworks powders. The method combines PCA for dimensionality reduction with linear regression for prediction, allowing assessment of how much of the spectral variation can be used to distinguish between the two classes. In this binary framework, ammunition samples were coded as 0 and fireworks samples as 1, with the regression model predicting a continuous value for each sample. A decision threshold of 0.5 was applied to convert predicted values into class assignments, enabling the derivation of a confusion matrix and classification metrics from the regression output.

**FIGURE 7:**
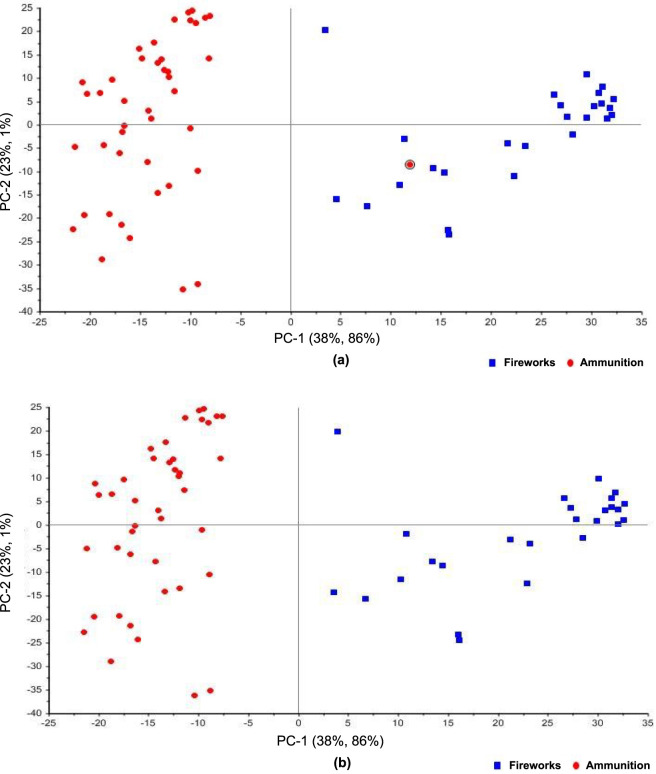
PCR score plots for FT-IR spectra of 69 gunpowder samples (43 ammunition, 26 fireworks) preprocessed using SNV. **(a)** Model including all samples, showing one ammunition sample (circled) projected within the fireworks cluster, indicating compositional overlap. **(b)** Model after exclusion of the overlapping sample, illustrating improved class separation and enhanced linear correlation between latent variables and sample categories.

Among the preprocessing techniques tested, SNV correction alone yielded the most consistent and interpretable results. SNV effectively minimized scattering effects and normalized intensity variations, producing a calibration R^2^ = 0.870 and a validation R^2^ = 0.864, with corresponding RMSE of 0.175 and 0.181, respectively. These results indicate that the major spectral variance captured by the principal components is chemically meaningful rather than noise-driven, consistent with earlier FT-IR chemometric studies on energetic materials (Banas et al., 2010; López-López and García-Ruiz, 2014; Silva et al., 2019). It should be noted, however, that R^2^ and RMSE were originally designed for continuous response variables and do not directly measure discriminative validity in a classification context. To complement these regression metrics, a confusion matrix was derived by applying the 0.5 threshold to the predicted values. Under the SNV-preprocessed PCR model including all samples, 42 of 43 ammunition samples and 26 of 26 fireworks samples were correctly assigned, yielding an ammunition sensitivity of 97.7%, a fireworks sensitivity of 100%, and an overall classification accuracy of 98.6%. The single misclassified ammunition sample is the one visible in the fireworks cluster in Figure 7a. These confusion-matrix-derived metrics confirm that the regression model achieves class discrimination comparable to, and in the case of fireworks sensitivity exceeding, the dedicated classifiers (LDA and SVM) reported above.

Inspection of the PCR score plot (Figure 7) revealed one ammunition sample projected within the fireworks cluster, suggesting spectral similarity rather than measurement error. This overlap likely reflects compositional convergence between certain propellant formulations and pyrotechnic mixtures that share oxidizing salts or organic binders, as previously reported in comparative studies of explosive residues (Banas et al., 2010; López-López and García-Ruiz, 2014). Although removing this misclassified sample improved regression metrics, raising the calibration R^2^ to 0.935 and the validation R^2^ to 0.924, with corresponding decreases in RMSE to 0.124 and 0.132, the spectral position of this point represents an authentic chemical ambiguity relevant to real forensic casework (Aljanaahi et al., 2025; Silva et al., 2019; Trache and Tarchoun, 2019). Its inclusion underscores that spectral similarity across energetic formulations can blur class boundaries, reinforcing the need for combined spectral-chemometric interpretation rather than purely statistical exclusion. The PCR model demonstrated strong predictive capability under minimal preprocessing.

### 3.6 PLS-R

PLS-R was applied to model the relationship between the FT-IR spectra and the categorical distinction between ammunition and fireworks samples. This approach captures the latent variables that best describe the covariance between predictor (spectral) and response (class) matrices, offering a robust alternative to purely variance-based methods such as PCR. As with the PCR model, ammunition samples were coded as 0 and fireworks samples as 1, and a decision threshold of 0.5 was applied to derive classification metrics from the continuous regression output.

**FIGURE 8:**
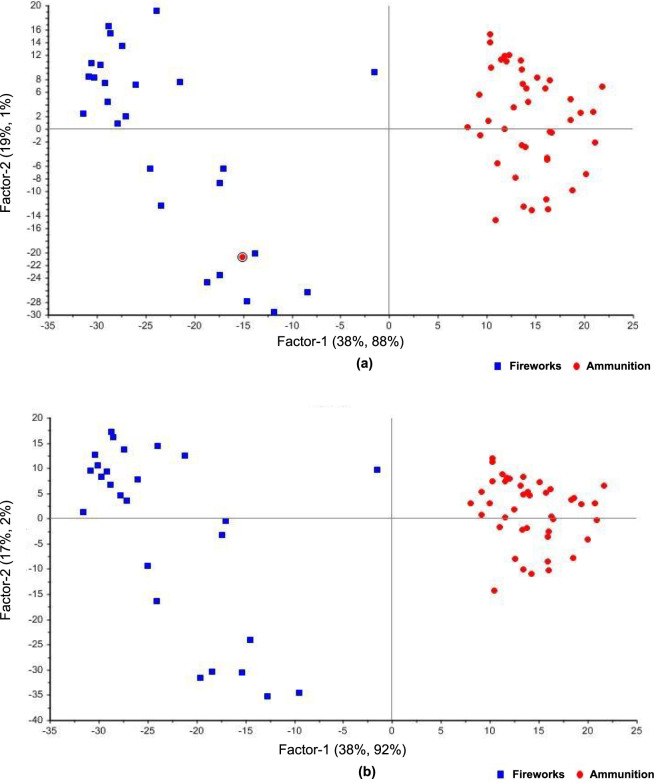
PLS-R score plots for FT-IR spectra of 69 gunpowder samples (43 ammunition, 26 fireworks) preprocessed using SNV. **(a)** Model including all samples, showing one ammunition sample projected within the fireworks cluster, indicating partial spectral overlap between the two classes. This is the same sample identified in the PCR analysis (Figure 7a). **(b)** Model after excluding the misclassified sample, demonstrating clearer class separation and improved linear correlation between latent variables and class identity.

Among all preprocessing configurations tested, SNV, normalization followed by SNV, and MSC followed by SNV yielded the highest predictive performance. The corresponding score plots are shown in Figure 8. The SNV-only preprocessing produced a calibration model that achieved a slope of 0.880, offset of 0.0747, RMSE of 0.168, and R^2^ of 0.880, while validation results yielded a slope of 0.851, offset of 0.0945, RMSE of 0.181, and R^2^ of 0.867. Applying the 0.5 decision threshold to the SNV-preprocessed model, 42 of 43 ammunition samples and 26 of 26 fireworks samples were correctly assigned, yielding an ammunition sensitivity of 97.7%, a fireworks sensitivity of 100%, and an overall classification accuracy of 98.6%. The single misclassified ammunition sample is the same specimen identified in the PCR analysis (Section 3.5), further confirming that this overlap reflects a consistent chemical ambiguity rather than model-specific instability.

Excluding this misclassified sample markedly improved calibration and validation regression metrics; R^2^ increased to 0.939 and 0.924, respectively, demonstrating that its removal enhanced model homogeneity and predictive precision. The spectral similarity responsible for the misclassification is likely linked to overlapping nitrate and nitrocellulose absorption bands common to both propellant and pyrotechnic formulations, consistent with the diagnostic profiles described in Figure 3.

The performance of other preprocessing configurations followed similar trends and are summarized in Table 3. Notably, the SNV and normalization followed by SNV configurations produced nearly identical metrics, suggesting that the addition of normalization prior to SNV does not alter the variance structure meaningfully when the dominant spectral variation is multiplicative scatter. In contrast, the MSC followed by SNV pipeline yielded lower R^2^ values (calibration: 0.827, validation: 0.787), consistent with the PCA findings (Section 3.2) that sequential application of two scatter-correction methods can redistribute variance in ways that reduce rather than enhance class discrimination. These results collectively highlight the importance of appropriate preprocessing and careful evaluation of misclassified cases, which may carry genuine chemical significance rather than representing random noise (Aljanaahi et al., 2025; Banas et al., 2010; López-López and García-Ruiz, 2014; Silva et al., 2019; Trache and Tarchoun, 2019).

**TABLE 3:** Calibration and validation results of PLS-R models using various preprocessing techniques, showing slope, offset, RMSE, and R^2^ values with and without the misclassified samples.

Preprocessing technique	Calibration slope	Calibration offset	Calibration RMSE	Calibration R^2^	Validation slope	Validation offset	Validation RMSE
SNV only	0.880	0.0747	0.168	0.880	0.851	0.0945	0.181
SNV only (misclassified removed)	0.939	0.0377	0.120	0.939	0.922	0.0510	0.132
Normalization → SNV	0.880	0.0747	0.168	0.880	0.852	0.0942	0.181
Normalization → SNV (misclassified removed)	0.939	0.0377	0.120	0.939	0.922	0.0510	0.132
MSC → SNV	0.830	0.108	0.202	0.827	0.790	0.134	0.220
MSC → SNV (misclassified removed)	0.940	0.0369	0.120	0.940	0.918	0.0535	0.131

### 3.7 SVR results

SVR was employed to evaluate the predictive relationship between the FT-IR spectral data and the categorical classes representing ammunition and fireworks compositions. The method has been increasingly applied in vibrational spectroscopy due to its ability to resolve nonlinear relationships between spectral descriptors and target variables, particularly in heterogeneous energetic materials (Aljanaahi et al., 2025; Banas et al., 2010; López-López and García-Ruiz, 2014; Silva et al., 2019). As with the PCR and PLS-R models (Sections 3.5 and 3.6), ammunition samples were coded as 0 and fireworks samples as 1, and a decision threshold of 0.5 was applied to derive classification metrics from the continuous regression output.

In this study, four kernel functions, linear, polynomial, RBF, and sigmoid, were compared under multiple preprocessing configurations to assess their effect on prediction accuracy. The RBF kernel produced the most consistent calibration and validation results across all conditions, confirming its suitability for datasets exhibiting nonlinear spectral variance. When combined with the baseline followed by normalization followed by SNV preprocessing sequence, the RBF model achieved the highest predictive performance (calibration R^2^ = 0.917, validation R^2^ = 0.888; Table 4). Applying the 0.5 decision threshold to this model, 42 of 43 ammunition samples and 26 of 26 fireworks samples were correctly assigned, yielding an ammunition sensitivity of 97.7%, a fireworks sensitivity of 100%, and an overall classification accuracy of 98.6%. The single misclassified ammunition sample is the same specimen identified across the PCR and PLS-R analyses, reinforcing that this overlap reflects a reproducible chemical ambiguity rather than a model-specific artifact. Identical RBF performance was obtained under SNV-only preprocessing (calibration R^2^ = 0.917, validation R^2^ = 0.888), indicating that baseline correction and normalization prior to SNV did not contribute additional discriminative variance for this kernel. Similar patterns have been observed in chemometric analyses of explosive residues, where nonlinear regression methods outperform linear approaches once spectral scattering and baseline drifts are corrected (Banas et al., 2010; López-López and García-Ruiz, 2014; Silva et al., 2019).

**TABLE 4:** SVR calibration and validation results for different kernels and preprocessing techniques, including RMSE and R^2^ values.

Kernel	Preprocessing technique	Calibration RMSE	Calibration R^2^	Validation RMSE	Validation R^2^
Linear	No preprocessing	0.411	0.481	0.446	0.381
Linear	Baseline → normalization → SNV	0.0463	0.991	0.265	0.735
Polynomial	Baseline → normalization → SNV	0.466	0.705	0.488	0.675
RBF	Baseline → normalization → SNV	0.140	0.917	0.163	0.888
Sigmoid	Baseline → normalization → SNV	0.166	0.887	0.179	0.868
RBF	SNV only	0.140	0.917	0.163	0.888

The influence of preprocessing was evident across all kernel types. Pipelines incorporating baseline correction and variance normalization markedly improved prediction stability, whereas models trained on unprocessed spectra showed higher residual errors (linear kernel without preprocessing: calibration R^2^ = 0.481, validation R^2^ = 0.381). The linear kernel under baseline followed by normalization followed by SNV preprocessing exhibited a notable discrepancy between calibration (R^2^ = 0.991) and validation (R^2^ = 0.735) performance, suggesting overfitting to the training data. This pattern is consistent with the high dimensionality of the spectral dataset relative to the sample size, where a linear model can fit the training set nearly perfectly but fails to generalize to unseen samples. By contrast, the RBF and sigmoid kernels maintained closer calibration-validation agreement, indicating better regularization of the spectral variance. The polynomial kernel yielded the weakest performance (calibration R^2^ = 0.705, validation R^2^ = 0.675), consistent with its poor classification performance observed in the SVM analysis (Section 3.4).

These outcomes demonstrate that the predictive capacity of SVR depends strongly on the interaction between kernel function and preprocessing strategy. Properly optimized spectral correction, particularly through the combination of baseline alignment and SNV scaling, enables the model to capture composition-dependent variance within the FT-IR dataset, yielding reliable discrimination between ammunition and fireworks samples (Aljanaahi et al., 2025; Banas et al., 2010; López-López and García-Ruiz, 2014; Silva et al., 2019).

## 4 Discussion

The discriminant behavior observed across all chemometric models arises from genuine chemical variance rather than mathematical artifacts. As established by the representative spectra in Section 3.1 (Figure 3), ammunition powders are dominated by nitrocellulose diagnostic bands at 1635, 1269, and 817 cm^−1^ (asymmetric NO_2_ stretch, symmetric NO_2_ stretch, and N-O deformation, respectively), supplemented by C-H stretching near 2916 cm^−1^ and C=O stretching near 1729 cm^−1^ (Larsson, 2014; Nunes et al., 2020). Fireworks powders, by contrast, exhibit oxidizer-dominated profiles featuring potassium perchlorate at 1029, 914, 618, and 464 cm^−1^ alongside potassium nitrate near 1399 cm^-1^ (Holló et al., 2019; Huang et al., 2023). The resulting class separation therefore reflects a fundamental compositional contrast between organic polymeric binders and inorganic oxidizer matrices, consistent with prior spectral interpretations of explosives and post-blast residues (Banas et al., 2010; Ewing and Kazarian, 2017; López-López and García-Ruiz, 2014; Zapata and García-Ruiz, 2021).

### 4.1 Influence of preprocessing order

Preprocessing sequence proved a critical determinant of classification performance. Pipelines beginning with baseline correction followed by normalization and SNV produced the clearest class separation, while reversed or disordered sequences degraded accuracy substantially; the most extreme case, SNV followed by normalization under LDA, reduced accuracy from 97.1% to 53.6% (Table 1). This behavior confirms that preprocessing is not a cosmetic adjustment but a chemically consequential step: baseline correction removes additive drift before normalization scales relative absorbances, and SNV eliminates multiplicative scatter, collectively preserving chemically meaningful variance (Silva et al., 2019; Sauzier et al., 2020; dos Santos et al., 2010; Aljanaahi et al., 2025). Reversing this order applies scatter correction to spectra still containing additive offsets, causing the correction to distort rather than stabilize the chemical signal. SNV alone yielded robust models for LDA, PCR, and PLS-R, supporting earlier reports that simple variance scaling can stabilize spectra dominated by particle-size and path-length effects (Silva et al., 2019; Trache and Tarchoun, 2019). The present benchmarking demonstrates, on real gunpowder samples, that the order of preprocessing operations is determinative for the recovery of chemically interpretable variance, a principle frequently noted but rarely quantified in explosives chemometrics.

### 4.2 Linear versus nonlinear model behavior

Linear and kernel-based models captured complementary dimensions of the data. The strong performance of LDA under SNV preprocessing (97.1% accuracy, with ammunition sensitivity of 97.7% and fireworks sensitivity of 96.2%; Table 1) indicates that much of the ammunition-fireworks distinction is linearly separable, governed by the relative intensities of nitrate, perchlorate, and nitrocellulose bands (Banas et al., 2010). SVM with RBF and sigmoid kernels matched this accuracy under the same preprocessing (Table 2), while additionally maintaining robust performance across a wider range of preprocessing configurations, reflecting the capacity of kernel methods to accommodate nonlinear variance arising from multi-oxidizer systems, metallic colorants, and stabilizer additives typical of fireworks mixtures (Aljanaahi et al., 2025; Banas et al., 2010; Sleiman et al., 2016).

The regression-based models (PCR, PLS-R, SVR) provided a complementary perspective. When their continuous outputs were converted to class assignments using a 0.5 decision threshold, all three achieved an overall classification accuracy of 98.6% under SNV preprocessing, correctly assigning 42 of 43 ammunition samples and 26 of 26 fireworks samples. This consistency across fundamentally different model architectures, discriminant, classifier, and regression, strengthens confidence that the observed performance reflects genuine chemical separability rather than algorithmic bias. The agreement also indicates that the R^2^ values reported for the regression models (0.864–0.888 for validation) underestimate the actual discriminative performance, since a single borderline prediction suffices to depress R^2^ while the confusion matrix remains near-perfect.

The SVR analysis further revealed that kernel selection interacts with model complexity. The linear SVR kernel exhibited pronounced overfitting under baseline followed by normalization followed by SNV preprocessing (calibration R^2^ = 0.991 versus validation R^2^ = 0.735; Table 4), whereas the RBF kernel maintained closer calibration-validation agreement (0.917 versus 0.888), consistent with its implicit regularization through the kernel width parameter. The polynomial kernel underperformed across both SVM and SVR analyses, indicating that its fixed-degree mapping is ill-suited to the variance structure of these spectral data.

### 4.3 Misclassification as chemical overlap

The single ammunition sample projected within the fireworks cluster was consistently identified across all regression models (PCR, PLS-R, and SVR) and in multiple LDA and SVM configurations, confirming that this overlap is reproducible and model-independent. Its spectral profile, examined in the context of the diagnostic bands identified in Section 3.1, likely exhibits enhanced intensity in the perchlorate or nitrate regions relative to typical ammunition samples, or reduced prominence of the nitrocellulose triplet, suggesting a propellant formulation that incorporates oxidizer components more characteristic of pyrotechnic mixtures. Comparable convergence between propellant and pyrotechnic signatures has been documented in FT-IR and post-blast residue analyses where certain formulations share common oxidizing salts or organic binders (Banas et al., 2010; López-López and García-Ruiz, 2014; Trache and Tarchoun, 2019; Zapata and García-Ruiz, 2021).

From a forensic perspective, such instances warrant interpretive caution: they represent transitional or hybrid formulations, not outliers to be discarded. Excluding the misclassified sample improved regression metrics substantially (e.g., PLS-R validation R^2^ from 0.867 to 0.924; Table 3), but this improvement reflects reduced chemical heterogeneity rather than correction of an error. Retaining the sample preserves an authentic representation of the compositional continuum that exists among real-world energetic materials.

### 4.4 Statistical comparison of model performance

Several preprocessing-model combinations achieved the same peak accuracy of 97.1% (LDA) or 98.6% (regression-derived), raising the question of whether performance differences among configurations are statistically meaningful. With only 69 samples and misclassification counts differing by one or two samples between the best-performing models, formal pairwise comparison tests such as McNemar’s test lack sufficient statistical power to detect significant differences. For example, comparing LDA under SNV (2 misclassifications) with LDA under normalization followed by SNV (also 2 misclassifications) yields identical contingency tables. Even comparing the best (2 errors) and moderately performing (3-4 errors) configurations produces marginal differences that would not reach significance at conventional thresholds given the small sample size. This observation does not diminish the practical utility of the results; rather, it indicates that multiple preprocessing-model combinations achieve near-equivalent performance, providing forensic laboratories with flexibility in implementation. Future studies employing larger sample sets would enable more discriminating statistical comparisons among models.

### 4.5 Forensic and analytical implications

The combined findings confirm that ATR-FT-IR coupled with optimized chemometric preprocessing can serve as a rapid, nondestructive, and legally defensible first-tier screening tool for classifying unknown energetic powders. The diagnostic spectral markers identified in this study, the nitrocellulose triplet at 1635, 1269, and 817 cm^−1^ for propellants and the perchlorate set at 1029, 914, 618, and 464 cm^−1^ together with nitrate at 1399 cm^−1^ for fireworks, directly correspond to functional groups relevant for forensic attribution (Banas et al., 2010; Banas et al., 2020; Ewing and Kazarian, 2017; López-López and García-Ruiz, 2014). The interpretability of these discriminant features strengthens evidentiary admissibility by linking statistical classification boundaries to known chemical structures (INTERPOL, 2019; Klapec et al., 2020; Zapata and García-Ruiz, 2021). Such models can guide confirmatory techniques, including ion chromatography for anion speciation, X-ray diffraction for crystalline oxidizers, or mass spectrometry for organic stabilizers (Forbes et al., 2020; Hutchinson et al., 2007; Schachel et al., 2020). Recent multi-method approaches integrating chromophoric reagents, SEM-EDS, and FTIR for shooting distance estimation (Yuksel et al., 2025), and elemental profiling of toxic versus lead-free primers using ICP-MS, SEM-EDS, and XPS (Yuksel et al.), demonstrate the broader forensic value of combining molecular and elemental techniques. Infrared imaging has further shown promise for visualizing residue distribution on complex substrates including dark, patterned, and biological surfaces (Yüksel et al., 2019), suggesting that the spectral-chemometric workflow developed here could complement imaging-based approaches in comprehensive forensic protocols.

Several limitations of the present study should be acknowledged. First, the dataset of 69 samples, although encompassing diverse commercial formulations, does not span the full range of international pyrotechnic products; extending the sample set to include imported brands, military-grade compositions, and post-blast residues would improve model generalization. Second, the absence of a fully independent external validation set means that the reported accuracies, while supported by stratified cross-validation, have not been confirmed on samples collected at different times or from different sources. Third, environmental CO_2_ absorption near 2349 cm^−1^ introduced minor secondary variance in the PCA loadings, suggesting that future work should employ purged enclosures or adaptive background correction to mitigate this interference (Ewing and Kazarian, 2017; Sauzier et al., 2020). Fourth, the class imbalance (43 ammunition versus 26 fireworks) could bias models toward the majority class; although the class-wise sensitivity analysis (Tables 1, 2) shows that both classes achieved high sensitivity under the best configurations, future studies should aim for more balanced sample distributions or explicitly incorporate class-weighting strategies.

Beyond spectral refinement, subsequent research should incorporate elemental profiling using complementary techniques such as ICP-MS, SEM-EDS, or XRF to characterize the metallic additives and oxidizer residues that drive many of the compositional differences between propellants and pyrotechnics (Yüksel et al., 2023; Aljanaahi et al., 2025; D’Uva et al., 2020). Integrating molecular (FT-IR) and elemental fingerprints within a unified chemometric framework would enable multi-modal classification and improve source attribution in forensic explosives analysis. Given its minimal sample preparation and fast spectral acquisition, the proposed workflow could be implemented within field-deployable FT-IR units for preliminary classification prior to confirmatory analysis.

### 4.6 Novelty and contribution

This work represents the first systematic benchmarking of ATR-FT-IR preprocessing pipelines for differentiating ammunition and fireworks powders, integrating both linear and nonlinear chemometric models under a unified validation strategy. Unlike previous studies that treated preprocessing as a fixed step, this research quantifies its order-specific impact on variance structure and classification fidelity. The addition of confusion-matrix-derived sensitivity metrics to the regression models (PCR, PLS-R, SVR) addresses a recognized limitation of using R^2^ alone for classification-converted regression tasks, providing forensic practitioners with directly interpretable performance indicators. The recognition of the misclassified sample as chemically plausible rather than erroneous, supported by its consistency across all model families, advances the interpretive rigor of forensic chemometrics by distinguishing compositionally hybrid spectra from true statistical anomalies. Collectively, these findings establish a transparent, reproducible workflow that bridges laboratory-level reliability with field-level speed, positioning ATR-FT-IR and chemometrics as a cornerstone technique for modern forensic explosives screening.

## 5 Conclusion

This study demonstrated that ATR-FT-IR spectroscopy combined with optimized chemometric modeling can reliably distinguish ammunition propellants from consumer fireworks powders. Across 69 samples (43 ammunition, 26 fireworks), class separation was driven by functional-group contrasts between the nitrocellulose diagnostic triplet (1635, 1269, 817 cm^−1^) in propellants and perchlorate-nitrate features (1029, 914, 618, 1399 cm^-1^) in fireworks. The systematic evaluation of preprocessing sequences confirmed that both the choice and order of preprocessing steps critically influence model performance; SNV-based pipelines consistently outperformed alternative configurations, while reversed sequences such as SNV followed by normalization reduced LDA accuracy to 53.6%.

LDA achieved 97.1% classification accuracy under SNV preprocessing, with class-wise sensitivities of 96.2% for fireworks and 97.7% for ammunition. SVM with RBF and sigmoid kernels matched this accuracy while offering greater adaptability to nonlinear spectral variance. Regression-based models (PCR, PLS-R, SVR) yielded validation R^2^ values of 0.864 to 0.888 and, when converted to classification via a 0.5 decision threshold, achieved 98.6% overall accuracy (fireworks sensitivity 100%, ammunition sensitivity 97.7%). A single ammunition sample was consistently projected within the fireworks cluster across all regression models, representing reproducible chemical overlap attributable to shared oxidizer and binder signatures rather than analytical error.

From a forensic perspective, the workflow offers a transparent, reproducible, and minimally destructive first-tier screening approach for unknown energetic powders. The discriminant features are directly traceable to known chemical functionalities, supporting admissibility under evidentiary standards that require scientifically interpretable results. Future research will expand the sample set to include a broader range of commercial pyrotechnic formulations and integrate elemental profiling techniques such as ICP-MS and XRF to complement molecular fingerprints, enabling multi-modal chemometric classification and improved source attribution in forensic explosives investigations.

## Data Availability

The original data are included in the article, and further inquiries can be directed to the corresponding author.

## References

[B1] Alblooshi R. A. Alremeithi R. H. Aljannahi A. H. Nahlé A. (2024). Comparative forensic discrimination of pink lipsticks using fourier transform infra-red and raman spectroscopy. Vib. Spectrosc. 130, 103640. 10.1016/j.vibspec.2023.103640

[B2] Aljanaahi A. Hakeem M. K. Aljanaahi A. Shah I. (2025). A review of analytical and chemometric strategies for forensic classification of homemade explosives. Anal. Sci. Adv. 6, e70010. 10.1002/ansa.70010 40242023 PMC12002426

[B3] Aljannahi A. Alblooshi R. A. Alremeithi R. H. Karamitsos I. Ahli N. A. Askar A. M. (2022). Forensic analysis of textile synthetic fibers using a FT-IR spectroscopy approach. Mol. Basel, Switz. 27 (13), 4281. 10.3390/molecules27134281 35807525 PMC9268719

[B4] Arce-Rubí S. Vargas-Ramírez S. Solís-Montiel E. Piedra-Marín G. Mora-Barrantes M. Carlos J. (2020). Identificación de explosivos Orgánicos en Indicios Post-Explosión mediante GC-MS y GC-NPD. Rev. Tecnol. Marcha 33 (3), 25–44. 10.18845/tm.v33i3.4377

[B5] Banas K. Banas A. Moser H. O. Bahou M. Li W. Yang P. (2010). Multivariate analysis techniques in the forensics investigation of the postblast residues by means of fourier transform-infrared spectroscopy. Anal. Chem. 82 (7), 3038–3044. 10.1021/ac100115r 20218700

[B6] Banas A. Banas K. Lo M. K. F. Kansiz M. Kalaiselvi S. M. P. Lim S. K. (2020). Detection of high-explosive materials within fingerprints by means of optical-photothermal infrared spectromicroscopy. Anal. Chem. 92 (14), 9649–9657. 10.1021/acs.analchem.0c00938 32567834

[B7] Baumgarten B. R. Huestis P. L. Cleveland A. H. Manner V. W. Freye C. E. (2023). New methods for trace analysis of gamma-irradiated pentaerythritol tetranitrate. Radiat. Phys. Chem. 212, 111143. 10.1016/j.radphyschem.2023.111143

[B8] Castro-Suarez J. R. Hernández-Rivera S. P. Pacheco-Londoño L. (2017). “Detection of primary and secondary explosives using infrared spectroscopy and chemometrics,” in Proceedings of the 15th LACCEI International Multi-Conference for Engineering, Education, and Technology: “Global Partnership for Development and Engineering Education,” (Latin American and Caribbean Consortium of Engineering Institutions), 81. 10.18687/LACCEI2017.1.1.81

[B9] Cetó X. O’Mahony A. M. Wang J. Del Valle M. (2013). Simultaneous identification and quantification of nitro-containing explosives by advanced chemometric data treatment of cyclic voltammetry at screen-printed electrodes. Talanta 107, 270–276. 10.1016/j.talanta.2012.12.042 23598222

[B10] Chauhan R. Kumar R. Diwan P. K. Sharma V. (2020). Thermogravimetric analysis and chemometric based methods for soil examination: application to soil forensics. Forensic Chem. 17, 100191. 10.1016/j.forc.2019.100191

[B25] de Oliveira Neves A. C. Soares G. M. de Morais S. C. da Costa F. S. L. Porto D. L. de Lima K. M. G. (2012). Dissolution testing of isoniazid, rifampicin, pyrazinamide and ethambutol tablets using near-infrared spectroscopy (NIRS) and multivariate calibration. J. Pharm. Biomed. Analysis 57, 115–119. 10.1016/j.jpba.2011.08.029 21908131

[B27] dos Santos E. O. Silva A. M. S. Fragoso W. D. Pasquini C. Pimentel M. F. (2010). Determination of degree of polymerization of insulating paper using near infrared spectroscopy and multivariate calibration. Vib. Spectrosc. 52 (2), 154–157. 10.1016/j.vibspec.2009.12.004

[B12] D’Uva J. A. DeTata D. May C. D. Lewis S. W. (2020). Investigations into the source attribution of party sparklers using trace elemental analysis and chemometrics. Anal. Methods 12, 4939–4948. 10.1039/D0AY01319F 33030194

[B13] Ewing A. V. Kazarian S. G. (2017). Infrared spectroscopy and spectroscopic imaging in forensic science. Analyst 142 (2), 257–272. 10.1039/C6AN02244H 27905577

[B14] Forbes T. P. Krauss S. T. Gillen G. (2020). Trace detection and chemical analysis of homemade fuel-oxidizer mixture explosives: emerging challenges and perspectives. Trends Anal. Chem. 131, 116023. 10.1016/j.trac.2020.116023 34135538 PMC8201619

[B16] Holló B. B. Petruševski V. M. Kovács G. B. Franguelli F. P. Farkas A. Menyhárd A. (2019). Thermal and spectroscopic studies on a double-salt-type pyridine–silver perchlorate complex having κ1-O coordinated perchlorate ions. J. Therm. Anal. Calorim. 138, 1193–1205. 10.1007/s10973-019-08663-1

[B17] Hu F. Wang L. J. Zhao W. Liu Y. C. Jing S. M. Liu P. (2021). Thermal decomposition kinetics and compatibility of 3, 5-Difluoro-2, 4, 6-Trinitroanisole (DFTNAN). Materials 14 (15), 4186. 10.3390/ma14154186 34361377 PMC8348359

[B46] Huang J. Zhang A. Xue H. Zhou J. Ding Y. Xiao Z. (2023). Biological treatment of nitrocellulose: investigation on structural and thermodynamic properties. Research Square (Preprint). 10.21203/rs.3.rs-3039202/v1

[B47] Hutchinson J. P. Evenhuis C. J. Johns C. Kazarian A. A. Breadmore M. C. Macka M. (2007). Identification of inorganic improvised explosive devices by analysis of postblast residues using portable capillary electrophoresis instrumentation and indirect photometric detection with a light-emitting diode. Analytical chemistry. 79 (18), 7005–7013. 10.1021/ac0708792 17705451

[B18] INTERPOL (2019). Review Papers: 19th INTERPOL International Forensic Science Managers Symposium. Lyon: INTERPOL.

[B19] Jalalvand A. R. (2021). Chemometrics in investigation of small molecule-biomacromolecule interactions: a review. Int. J. Biol. Macromol. 181, 478–493. 10.1016/j.ijbiomac.2021.03.184 33798569

[B20] Klapec D. J. Czarnopys G. Pannuto J. (2020). Interpol review of detection and characterization of explosives and explosives residues 2016-2019. Forensic Sci. Int. Synergy 2, 670–700. 10.1016/j.fsisyn.2020.01.020 33385149 PMC7770463

[B21] Larsson A. (2014). Chemical characterisation of nitrocellulose. (thesis). Örebro University, Örebro, Sweden. Available online at: https://www.diva-portal.org/smash/record.jsf?pid=diva2%3A781469 (Accessed October 15, 2025).

[B22] López-López M. García-Ruiz C. (2014). Infrared and raman spectroscopy techniques applied to identification of explosives. Trends Anal. Chem. 54, 36–44. 10.1016/j.trac.2013.10.011

[B23] Nunes S. Ramacciotti F. Neves A. Angelin E. M. Ramos A. M. Roldão É. (2020). A diagnostic tool for assessing the conservation condition of cellulose nitrate and acetate in heritage collections: quantifying the degree of substitution by infrared spectroscopy. Npj Herit. Sci. 5 (1), 1–11. 10.1186/s40494-020-00373-4

[B26] Rismiwandira K. Roosmayanti F. Pahlawan M. F. R. Masithoh R. E. (2021). “Application of fourier transform near-infrared (FT-NIR) spectroscopy for detection of adulteration in palm sugar,” in IOP Conference Series: Earth and Environmental Science (Bristol, United Kingdom: IOP Publishing). 10.1088/1755-1315/653/1/012122

[B28] Sauzier G. van Bronswijk W. Lewis S. W. (2020). Chemometrics in forensic science: approaches and applications. Forensic Chem. 20, 100264–102448. 10.1039/D1AN00082A 33729240

[B29] Schachel T. D. Stork A. Schulte-Ladbeck R. Vielhaber T. Karst U. (2020). Identification and differentiation of commercial and military explosives Via high performance liquid chromatography-high resolution mass spectrometry (HPLC-HRMS), X-ray diffractometry (XRD) and X-ray fluorescence spectroscopy (XRF): towards a forensic substance database on explosives. Forensic Sci. Int. 308, 110180. 10.1016/j.forsciint.2020.110180 32059131

[B30] Serol M. Ahmad S. M. Quintas A. Família C. (2023). Chemical analysis of gunpowder and gunshot residues. Molecules 28, 5550. 10.3390/molecules28145550 37513421 PMC10386329

[B31] Sharma B. Gadi R. (2023). Analytical tools and methods for explosive analysis in forensics: a critical review. Crit. Rev. Anal. Chem. 0 (0), 1–27. 10.1080/10408347.2023.2274927 37934616

[B32] Shrivastava P. Jain V. K. Nagpal S. (2021). Gunshot residue detection technologies–a review. Egypt J. Forensic Sci. 11, 11. 10.1186/s41935-021-00223-9

[B33] Silva C. Braz A. Pimentel M. F. (2019). Vibrational spectroscopy and chemometrics in forensic chemistry: critical review, current trends and challenges. J. Braz. Chem. Soc. 10.21577/0103-5053.20190140

[B34] Sleiman J. B. Engelbrecht S. Merlat L. Fischer B. Bousquet B. Mounaix P. (2016). “Chemometrics applied to terahertz and raman spectra for explosives analysis,” in 2016 41st International Conference on Infrared, Millimeter, and Terahertz Waves (IRMMW-THz) (IEEE), 1–2. 10.1109/IRMMW-THz.2016.7758801

[B35] Sun W. Gao X. Wang Y. Tong Y. (2022). Thermal safety analysis of On-Site emulsion explosives mixed with waste engine oil. Energies 15 (3), 895. 10.3390/en15030895

[B36] Trache D. Tarchoun A. F. (2019). Differentiation of stabilized nitrocellulose during artificial aging: spectroscopy methods coupled with principal component analysis. J. Chemom. 33 (6), e3163. 10.1002/cem.3163

[B11] van Damme I. M. Mestres-Fitó P. Ramaker H. J. Hulsbergen A. W. C. van der Heijden A. E. D. M. Kranenburg R. F. (2023). Rapid and On-Scene chemical identification of intact explosives with portable near-infrared spectroscopy and multivariate data analysis. Sensors 23 (8), 3804. 10.3390/s23083804 37112149 PMC10146942

[B37] Wang L. Sun H. Li Q. Fei B. Pan R. Zhou X. (2025). Exploration of the combustion characteristic based on the pyrolysis and combustion spectral analysis of single base propellant. Thermochim. Acta 743, 179901. 10.1016/j.tca.2024.179901

[B38] Wolny P. Tuśnio N. Lewandowski A. Mikołajczyk F. Kuberski S. (2021). Self-acting formation of an ANFO similar type of explosive under fire conditions: a case study. Energies 14 (21), 6980. 10.3390/en14216980

[B39] Yao F. Xu P. Jia H. Li X. Yu H. Li X. (2022). Thermogravimetric analysis on a resonant microcantilever. Anal. Chem. 94 (26), 9380–9388. 10.1021/acs.analchem.2c01374 35731930

[B40] Yüksel B. Şen N. Ögünç G. I. Erdoğan A. (2023). Elemental profiling of toxic and modern primers using ICP-MS, SEM-EDS, and XPS: an application in firearm discharge residue investigation. Aust. J. Forensic Sci. 55, 529–546. 10.1080/00450618.2022.2043436

[B41] Yüksel B. Ho M. Ovide O. Pyl C. V. Trejos T. (2019). Infrared imaging as a complementary aid in estimating muzzle-to- target shooting distance: an application on dark, patterned and bloody sample. Turkiye Klinikleri J. Foren. Sci. Leg. Med. 16, 73–80. 10.5336/forensic.2019-64837

[B42] Yuksel B. Sen N. Şeker M. E. Ogunc G. İ. Bulut S. (2025). Integrating chromophoric and instrumental methods (SEM-EDS and FTIR) for accurate shooting distance estimation: an inter-laboratory study in forensic chemistry. CAC 21, 1333–1344. 10.2174/0115734110352073241122164830

[B43] Zapata F. García-Ruiz C. (2021). Chemical classification of explosives. Crit. Rev. Anal. Chem. 51 (7), 656–673. 10.1080/10408347.2020.1760783 32397736

[B44] Zeman S. Gazda Š. Štolcová A. Dráb J. (1993). New correlations of the thermogravimetric analysis data of some commercial explosives. Thermochim. Acta 230, 177–189. 10.1016/0040-6031(93)80359-I

[B45] Zhou J. Yu L. Ding Q. Wang R. (2019). Textile fiber identification using near-infrared spectroscopy and pattern recognition. AUTEX Res. J. 19 (2), 201–209. 10.1515/aut-2018-0055

