# Correction: Forensic classification of gunpowder and fireworks powders by ATR-FT-IR spectroscopy and chemometric modelling

**DOI:** 10.3389/fchem.2026.1848505

**Published:** 2026-05-22

**Authors:** Abdulrahman Aljanaahi, Abdulla Aljanaahi, Noora Abdulkarim Ahli, Roudha Alblooshi, Abdulla Yasin, Iltaf Shah

**Affiliations:** 1 Dubai Police General Headquarters, Dubai, United Arab Emirates; 2 Department of Chemistry, College of Science, UAE University, Al Ain, United Arab Emirates

**Keywords:** energetic materials, explosives, machine learning, multivariate data analysis, preprocessing optimisation, spectral preprocessing

The reference for Castro-Suarez et al. (2017) was erroneously written as:

Castro-Suarez, J. R. (2017). “Detection of primary and secondary explosives using infrared spectroscopy and chemometrics,” in Global Partnerships for Development and Engineering Education: Proceedings of the 15th LACCEI International Multi-Conference for Engineering, Education and Technology, July 19-21, 2017 (Boca Raton, FL, United States), 81. Available online at: https://dialnet.unirioja.es/servlet/articulo?codigo=7353048 (Accessed October 15, 2025).

It should be:

“Castro-Suarez, J. R., Hernández-Rivera, S. P., and Pacheco-Londoño, L. (2017). “Detection of Primary and Secondary Explosives Using Infrared Spectroscopy and Chemometrics,” in *Proceedings of the 15th LACCEI International Multi-Conference for Engineering, Education, and Technology: “Global Partnership for Development and Engineering Education,”* (Latin American and Caribbean Consortium of Engineering Institutions), 81. doi: 10.18687/LACCEI2017.1.1.81.”

The in-text citations should therefore be “Castro-Suarez et al., 2017” instead of “Castro-Suarez, 2017”.

The reference for D’Uva et al. (2020) was erroneously written as:

D’Uva, J. (2022). Source attribution of party sparklers using ICP-MS and chemometrics. *Forensic Chem.* 27, 100377. doi:10.1039/D0AY01319F.

It should be:

“D’Uva, J. A., DeTata, D., May, C. D., and Lewis, S. W. (2020). Investigations into the source attribution of party sparklers using trace elemental analysis and chemometrics. *Anal. Methods* 12, 4939–48. doi: 10.1039/D0AY01319F.”

The in-text citations should therefore be “D’Uva et al., 2020” instead of “D’Uva, 2022”.

The reference for Holló et al. (2019) was erroneously written as:

Holló, B. B., Petruševski, V. M., Kovács, G. B., Franguelli, F. P., Farkas, A., and Menyhárd, A., (2019). Thermal and spectroscopic studies on a double-salt-type pyridine–silver perchlorate complex having κ1-O coordinated perchlorate ions.

It should be:

“Holló, B. B., Petruševski, V. M., Kovács, G. B., Franguelli, F. P., Farkas, A., Menyhárd, A., et al. (2019). Thermal and spectroscopic studies on a double-salt-type pyridine–silver perchlorate complex having κ1-O coordinated perchlorate ions. *J. Therm. Anal. Calorim*. 138, 1193–1205. doi: 10.1007/s10973-019-08663-1.”

The reference for INTERPOL (2019) was erroneously written as:

Interpol (2018). Interpol review of explosives and explosive devices. *Interpol Tech. Rep*.

It should be “INTERPOL (2019). *Review Papers: 19th INTERPOL International Forensic Science Managers Symposium*. Lyon: INTERPOL.”

The in-text citations should therefore be “INTERPOL, 2019” instead of “Interpol, 2018”.

The reference for Serol et al. (2023) was erroneously written as:

Serol, M., Esteve-Turrillas, F. A., Guardia, M., and García-Ruiz, C. (2023). Chemical analysis of gunpowder and gunshot residues. *Molecules* 28 (14), 5550. doi:10.3390/molecules28145550.

It should be:

“Serol, M., Ahmad, S. M., Quintas, A., and Família, C. (2023). Chemical analysis of gunpowder and gunshot residues. *Molecules* 28, 5550. doi: 10.3390/molecules28145550.”

The reference for Shrivastava et al. (2021) was erroneously written as:

Shrivastava, P., and Sharma, N. (2021). Gunshot residue detection technologies—A review. *Egypt. J. Forensic Sci.* 11, 1–20. doi:10.1186/s41935-021-00223-9.

It should be:

“Shrivastava, P., Jain, V. K., and Nagpal, S. (2021). Gunshot residue detection technologies—a review. *Egypt J. Forensic Sci*. 11, 11. doi: 10.1186/s41935-021-00223-9.”

The in-text references should therefore be “Shrivastava et al., 2021” instead of “Shrivastava and Sharma, 2021”.

The reference for Silva et al. (2019) was erroneously written as:

Silva, C. S., Braz, A., and Pimentel, M. F. (2019). Vibrational spectroscopy and chemometrics in forensic chemistry: critical review, current trends and challenges. *J. Braz. Chem. Soc*. 30 (11), 2291–2298. doi:10.21577/0103-5053.20190140.

It should be:

“Silva, C., Braz, A., and Pimentel, M. F. (2019). Vibrational spectroscopy and chemometrics in forensic chemistry: critical review, current trends and challenges. *J. Braz. Chem. Soc.* doi: 10.21577/0103-5053.20190140.”

The reference for Yüksel et al. (2019) was erroneously written as:

Yuksel, B., Ozler-Yigiter, A., Bora, A., and Acar, T. (2019). Analysis of gunshot residue patterns on dark-colored, patterned, and bloody textiles using infrared imaging. *J. Forensic Sci*. 64 (5), 1466–1473. doi:10.5336/forensic.2019-64837.

It should be:

“Yüksel, B., Ho, M., Ovide, O., Pyl, C. V., and Trejos, T. (2019). Infrared imaging as a complementary aid in estimating muzzle-to-target shooting distance: an application on dark, patterned and bloody sample. *Turkiye Klinikleri J. Foren. Sci. Leg. Med.* 16, 73–80. doi: 10.5336/forensic.2019-64837.”

The reference for Yüksel et al. (2023) was erroneously written as:

Yuksel, B., Nic Daeid, D., and Yenigul, A. B. (2022). Elemental profiling of ammunition primers by ICP-MS, SEM-EDS, and XPS: discrimination of toxic and lead-free compositions. *Forensic Chem*. 30, 100436. doi:10.1080/00450618.2022.2043436.

It should be:

“Yüksel, B., Şen, N., Ögünç, G. I., and Erdoğan, A. (2023). Elemental profiling of toxic and modern primers using ICP-MS, SEM-EDS, and XPS: an application in firearm discharge residue investigation. *Aust. J. Forensic Sci.* 55, 529–546. doi: 10.1080/00450618.2022.2043436.”

The in-text citations should therefore be “Yüksel et al., 2023” instead of “Yuksel et al., 2022”.

The reference for Yuksel et al. (2025) was erroneously written as:

Yuksel, B., Ozler-Yigiter, A., and Acar, T. (2025). Estimation of shooting distance by integrating chromophoric test, SEM-EDS, and FT-IR analysis with multivariate statistical methods. *Forensic Sci. Int.* 366, 112280. doi:10.2174/0115734110352073241122164830.

It should be:

“Yuksel, B., Sen, N., Şeker, M. E., Ogunc, G. İ., and Bulut, S. (2025). Integrating chromophoric and instrumental methods (SEM-EDS and FTIR) for accurate shooting distance estimation: an inter-laboratory study in forensic chemistry. *CAC* 21, 1333–1344. doi: 10.2174/0115734110352073241122164830.”

The original article has been updated.

